# Differential Patterns of Domain-Specific Cognitive Complaints and Awareness Across the Alzheimer’s Disease Spectrum

**DOI:** 10.3389/fnagi.2022.811739

**Published:** 2022-06-16

**Authors:** Federica Cacciamani, Valérie Godefroy, Simona M. Brambati, Raffaella Migliaccio, Stéphane Epelbaum, Maxime Montembeault

**Affiliations:** ^1^ARAMISLab, Inserm, CNRS, Sorbonne Université, Inria, Paris Brain Institute (ICM), Pitié-Salpêtrière Hospital, Paris, France; ^2^PHARes Team, Bordeaux Population Health, University of Bordeaux, Inserm, Bordeaux, France; ^3^FrontLab, Paris Brain Institute (ICM), Pitié-Salpêtrière Hospital, Paris, France; ^4^Department of Psychology, University of Montreal, Montreal, QC, Canada; ^5^Centre de recherche de l’Institut Universitaire de Gériatrie de Montréal, Montréal, QC, Canada; ^6^Institute of Memory and Alzheimer’s disease (IM2A), Pitié-Salpêtrière Hospital, Paris, France; ^7^Douglas Mental Health University Institute, Montreal, QC, Canada; ^8^Department of Psychiatry, McGill University, Montreal, QC, Canada; ^9^Memory & Aging Center, Department of Neurology, University of California, San Francisco, San Francisco, CA, United States

**Keywords:** awareness, metacognition, anosognosia, Alzheimer’s disease, language, executive function, memory, visuospatial abilities

## Abstract

**Background**: Characterizing self- and informant-reported cognitive complaints, as well as awareness of cognitive decline (ACD), is useful for an early diagnosis of Alzheimer’s disease (AD). However, complaints and ACD related to cognitive functions other than memory are poorly studied. Furthermore, it remains unclear which source of information is the most useful to distinguish various groups on the AD spectrum.

**Methods**: Self- and informant-reported complaints were measured with the Everyday Cognition questionnaire (ECog-Subject and ECog-StudyPartner) in four domains (memory, language, visuospatial, and executive). ACD was measured as the subject-informant discrepancy in the four ECog scores. We compared the ECog and ACD scores across cognitive domains between four groups: 71 amyloid-positive individuals with amnestic AD, 191 amnestic mild cognitive impairment (MCI), or 118 cognitively normal (CN), and 211 amyloid-negative CN controls, selected from the ADNI database. Receiver operating characteristic curves analysis was performed to evaluate the accuracy of the ECog and ACD scores in discriminating clinical groups.

**Results**: Self- and informant-reported complaints were generally distributed as follows: memory, language, executive, and visuospatial (from the most severe to the least severe). Both groups of CN participants presented on average more memory and language complaints than their informant. MCI participants showed good agreement with their informants. AD participants presented anosognosia in all domains, but especially for the executive domain. The four ECog-StudyPartner sub-scores allowed excellent discrimination between groups in almost all classifications and performed significantly better than the other two classifiers considered. The ACD was excellent in distinguishing the participants with AD from the two groups of CN participants. The ECog-Subject was the least accurate in discriminating groups in four of the six classifications performed.

**Conclusion**: In research, the study of complaint and anosognosia should not be reduced solely to the memory domain. In clinical practice, non-amnestic complaints could also be linked to Alzheimer’s disease. The presence of an informant also seems necessary given its accuracy as a source of information.

## Introduction

In the past, Alzheimer’s disease (AD) was clinically defined as a dementia syndrome (McKhann et al., 1984). The arrival of biomarkers has allowed a more accurate description of its pre-dementia stages. Technical and scientific progress has made it possible to develop increasingly precise diagnostic techniques. They allow to visualize the patient’s brain *in vivo* and to measure pathological hallmarks of AD, such as amyloid and tau pathology, and its neurodegenerative processes. We now know that years pass before neuropathology causes cognitive changes (i.e., *preclinical AD*, Dubois et al., 2010). The disease begins to manifest with a *transitional* or *subtle* cognitive decline (Sperling et al., 2011; Jack et al., 2018), meaning that performance is below the individual’s baseline cognitive level, although neuropsychological scores are not yet considered pathological. This condition precedes *mild cognitive impairment* (MCI, Albert et al., 2011), also known as *prodromal AD* (Dubois et al., 2010), which is instead detectable by neuropsychological testing, and which in turn precedes dementia.

One of the central pieces of information used to establish a diagnosis on the AD spectrum is the report of cognitive complaints from both the patient and the informant, which are usually collected during the initial clinical interview. History-taking from the patient and a knowledgeable informant is necessary, as stated in the current diagnostic criteria for dementia due to AD (McKhann, 2011), mild cognitive impairment (Albert et al., 2011), and subjective cognitive decline (Jessen et al., 2020). Therefore, the characterization of the cognitive complaints typical of patients with early-stage AD is one of the most studied topics to better understand the pre-dementia stages and for better early detection of AD (Jessen et al., 2020). In fact, the report of a cognitive complaint is one of the few ways that individuals with early neurodegeneration come to medical attention (Stewart et al., 2010). Cognitive-complainers are more likely to have abnormal biomarkers consistent with AD pathology, e.g., increased amyloid deposition (Perrotin et al., 2012), decreased metabolism (Mosconi et al., 2008), and cortical atrophy (Saykin et al., 2006). However, it is also a condition known to be nonspecific, with a high prevalence in the general population (Condret-Santi, 2013). Therefore, investigating the cognitive difficulties reported by a family member or close friend has also been studied for this purpose, and appears to be a particularly useful indicator of AD pathology (Gavett, 2011; Brunet et al., 2019), as well as for diagnostic accuracy (Gifford, 2015). The combination of self- and informant-reported cognitive complaints can also inform about the awareness of cognitive decline (ACD), which is another crucial information for individuals on the AD spectrum. Recent studies have shown that patients with early-stage AD may already present with reduced ACD (Hanseeuw et al., 2020), leading, in most cases (Turró-Garriga et al., 2016), to overt anosognosia in late-stage AD. The index of ACD, calculated for example as the difference between self- and informant-reports (Cacciamani et al., 2017, 2020), could provide added value for assessing the risk of AD in an individual, and function as a good predictor of future decline.

Due to the high frequency of amnestic AD dementia, research in the field of cognitive complaints and awareness is highly focused on episodic memory (Gagliardi et al., 2020; Jessen et al., 2020). In this context, non-amnestic cognitive complaints are less studied, but still of interest. First, patients or their families also report difficulties other than memory problems, such as language complaints or difficulty retrieving words (Rohrer, 2008; Montembeault et al., 2022), executive functioning (Valech et al., 2018), and visuospatial complaints (Mendez, 1990). Secondly, recent studies have highlighted the relevance of non-amnestic cognitive complaints in patients on the AD spectrum.

For example, in cognitively unimpaired individuals, word-finding complaints are as frequent and severe as memory complaints, and these complaints are more frequent and severe than executive and visuospatial complaints (Montembeault et al., 2022). Furthermore, self-reported cognitive complaints in language and executive function domains have been shown to help in distinguishing cognitively-normal amyloid-negative and amyloid-positive controls (La Joie et al., 2016; Valech et al., 2018; Montembeault et al., 2022). Shokouhi et al. (2019) investigated whether domain-specific complaints were equally or differently associated with amyloid and tau pathology in a group of cognitively-normal elderly individuals. They found that planning and visuospatial complaints were primarily associated with tau pathology, while memory and organizational complaints were primarily associated with amyloid deposits. This suggests that domain-specific complaints can be subtended by different processes (Shokouhi et al., 2019). Nonetheless, additional evidence across the full AD spectrum is needed to fully establish if complaints and awareness of non-amnestic domains (language, visuospatial, executive) are clinically useful. Anosognosia is also a multidimensional construct (Bertrand et al., 2019; Mayelle et al., 2022). In Bertrand and collaborators, patients with AD presented anosognosia regarding their overall medical condition and executive disorders, but they were well aware of their levels of disinhibition and apathy (Bertrand et al., 2019). Another study has shown more severe anosognosia for memory and activities of daily living alterations in patients with dementia, but an under-estimation of function in the socio-emotional domain (Marková et al., 2014).

While the clinical relevance of self- and informant-reported cognitive complaints and ACD have been shown, only a very few studies have investigated which piece of information is the most useful in distinguishing individuals at different stages on the AD spectrum. A study by Rueda (2015) compared the utility of informant- and self-report of cognitively-relevant functional abilities to discriminate diagnostic groups across the AD spectrum. They found that informants’ complaints were systematically more accurate than self-reports in distinguishing different stages of the disease, and that informant-report was consistently more associated with objective markers of the disease than self-reports, although self-reported functional status may still have some utility in early disease. However, they did not compare the respective utilities of informant- and self-report with the utility of ACD to predict the stage of the disease. Besides, they only used a global score of cognitive abilities (ECog total score), without considering the predictive values for each cognitive domain. Answering these questions could guide researchers and clinicians on the most optimal measures to use to distinguish these populations, both in terms of sources of information and specific cognitive domains.

In this study, we measured self-reported cognitive complaints, informant-reported complaints, and ACD across four cognitive domains (memory, language, visuospatial, executive) and between 71 amyloid-positive individuals with amnestic AD, 191 amnestic mild cognitive impairment (MCI) or 118 cognitively normal (CN), and 211 amyloid-negative CN controls from the ADNI database. Our first objective was to compare the intensity of self-reported complaints, informant-reported complaints, and ACD, by cognitive domain across the AD spectrum. We hypothesize that while episodic memory complaints will be the most elevated in all groups, non-amnestic cognitive complaints, especially language and executive complaints, will also distinguish the different groups on the AD spectrum and therefore be useful clinically. Furthermore, we expect AD patients to present anosognosia in all cognitive domains, but especially in memory and executive functioning. Our second objective was to measure how accurately self- and informant-reported complaints and ACD (i.e., subject-informant discrepancy) in the four investigated cognitive domains can discriminate the four clinical groups. We hypothesize that informant-reported cognitive complaints and ACD will be better than self-reported complaints in distinguishing clinical groups. Furthermore, demonstrating that informant-reported complaints and ACD in all cognitive domains allow for a good prediction of clinical groups will underline the clinical significance of investigating non-amnestic domains even in amnestic MCI and AD.

## Materials and Methods

### Participants

Data used in the preparation of this article was obtained from the Alzheimer’s Disease Neuroimaging Initiative (ADNI) database (adni.loni.usc.edu). The ADNI was launched in 2003 as a public-private partnership, led by Principal Investigator Michael W. Weiner, MD. The primary goal of ADNI has been to test whether serial magnetic resonance imaging (MRI), positron emission tomography (PET), other biological markers, and clinical and neuropsychological assessment can be combined to measure the progression of MCI and early AD. For up-to-date information, see www.adni-info.org.

We selected four groups of participants: amyloid-positive (Aβ+) individuals diagnosed with AD, MCI, or cognitively-normal (CN) at baseline, and amyloid-negative (Aβ−) healthy controls. Participants were considered Aβ+ when they had at least one positive amyloid marker. Amyloid markers considered were ^8^F-AV-45 PET [positive if retention ratio >1.1 (Landau, 2013)], PiB-PET [positive if retention ratio >1.5 (Donohue et al., 2014)], and CSF [positive if β amyloid level <192 pg/ml (Donohue et al., 2014)] No restrictions were imposed based on their cognitive status. We included Aβ+ subjects with normal cognition (i.e., subjects at risk of preclinical AD), with a diagnosis of MCI (or *prodromal AD*), or with a diagnosis of AD. The group of healthy controls consisted of cognitively unimpaired individuals who presented a negative status to all three amyloid markers considered, using the same reference values indicated above.

The CN status was reserved for participants with normal memory on the Wechsler Memory Scaled - Revised (WMS-R) Logical Memory II (LM II) test, Mini-Mental State Examination (MMSE) score between 24 and 30 (inclusive), Clinical Dementia Rating (CDR) = 0, and without significant impairment in activities of daily living. There was no criterion regarding memory complaints. Participants were classified as MCI if they presented subjective memory concerns as reported by the subject, their study-partner or clinician, had abnormal memory function on the WMS-R LM II test, an MMSE score between 24 and 30 (inclusive) and a CDR score = 0.5. Their general cognition and functional performance were sufficiently preserved so that a diagnosis of AD could not be made. Diagnosis of AD was made in participants with a memory complaint confirmed by a study-partner (or reported only by the study-partner), with abnormal memory on the WMS-R LM II test, with an MMSE score between 20 and 26 (inclusive), with a CDR score = 0.5 or 1, and who met the NINCDS/ADRDA criteria for probable AD.

All participants were aged between 55 and 90 years (inclusive), had completed a minimum of six degrees of education and did not have vascular dementia, depression, sensory disturbances, or other medical conditions that could interfere with the study. A study-partner who had frequent contact with the participant (for example, an average of 10 h per week or more) also accompanied him/her to visits and filled out questionnaires. We selected only participants with a maximum of one missing observation per cognitive domain for self- and informant-reported complaints (i.e., only subjects with a maximum of 10% missing data).

### Subjective Measures of Cognitive Decline

Subjects and study-partners independently completed two parallel versions of the Everyday Cognition questionnaire (ECog-Subject and ECog-StudyPartner; Farias, 2008), which asks to compare the subject’s current cognitive efficiency with that of 10 years ago. Four areas are assessed: Memory (eight items, for example, “Remembering a few shopping items without a list”), Language (nine items, for example, “Forget the name of objects”), Visuospatial ability (seven items, for example, “Follow a map to find a new location”) and Executive functions (15 items from the planning, organization, and divided attention sub-scales, for example, “Plan a sequence of stops on a shopping trip”). Answers are on a 4-point scale from 1 (“No change or performs better than 10 years ago”) to 4 (“Performs task much worse than 10 years ago”). The ECog-Subject and ECog-StudyPartner scores were calculated by averaging the responses on the items related to each cognitive domain, with a possible range between 1 and 4. We also calculated a global score for the Ecog-Subject and Ecog-StudyPartner by averaging the four domains.

### Awareness of Cognitive Decline (ACD)

As a measure of ACD, we used the subject-informant discrepancy (ECog Subject *minus* ECog-StudyPartner), which we calculated separately for each of the four ECog sub-scales. This resulted in four measures of awareness of changes in memory, language, visuospatial, and executive functions, respectively. We also calculated a global score for the ACD by averaging the four domains. The awareness scores ranged from −3 to 3. A score of zero indicates perfect agreement between the subject and the study-partner. A score of −3 indicates complete anosognosia (i.e., ECog-Subject >Ecog - StudyPartner). A score of 3 indicates an intense cognitive complaint not confirmed by the study-partner (i.e., ECog-Subject <Ecog-StudyPartner).

### Cognitive Scores

We used the MMSE as a global measure of cognitive functioning. As objective measures of memory, language, executive function and visuospatial abilities, we used four cognitive composites developed from the ADNI neuropsychological battery using item response theory. The memory composite included the Rey Auditory Verbal Learning Test, AD Assessment Schedule - Cognition (ADAS-Cog) memory items, MMSE memory items, and Logical Memory (Crane et al., 2012). The language composite included the Boston Naming Test, Category Fluency—animals, Category Fluency—vegetables, ADAS-Cog language items, MMSE language items, and MoCA language items (Choi et al., 2020). The visuospatial composite included the Clock drawing test, ADAS-Cog language items, and MMSE language items (Choi et al., 2020). The executive function composite included Category Fluency—animals, Category Fluency—vegetables, Trails A and B, Digit span backward, WAIS-R Digit Symbol Substitution, and five Clock Drawing items (circle, symbol, numbers, hands, time; Gibbons et al., 2012).

### Statistical Analysis

Statistical analyses were performed using RStudio (version 1.2.5033, RStudio, Inc) and IBM SPSS Statistics, Armonk, NY (version 26.0.0.1). Missing observations in ECog items were systematically imputed when a maximum of one response was missing per subscale (i.e., per cognitive domain), which represents a maximum of 10% of items per subject. Missing observations were imputed by the mean score of all other items of the subscale.

#### Study Population

We used χ^2^ test for categorical variables and one-way ANOVA (with Tukey correction) for continuous variables to compare demographical and clinical data between clinical groups.

#### Objective 1: Comparison of ECog-Subject, ECog-StudyPartner, and ACD Between Cognitive Domains and Clinical Groups

We used a mixed ANOVA design to test the main and interaction effects of the clinical group (between-subjects factor) and cognitive domain (within-subjects factor) on the eight ECog scores (four ECog-Subject, four ECog-StudyPartner) and the four anosognosia scores, controlling for age, sex, and education. To explore significant effects, we performed *post-hoc* comparisons using one-way ANOVA followed by pairwise *t*-tests with Bonferroni correction for multiple comparisons.

#### Objective 2: Accuracy of Domain-Specific ECog-Subject, ECog-StudyPartner, and ACD in Discriminating the Four Groups

Receiver operating characteristic curves (ROC) and the nonparametric estimate of the area under the ROC (AUC) based on the trapezoidal rule were used to evaluate the accuracy of predicting clinical groups using the ECog-Subject, ECog-StudyPartner, and ACD measures by domain (Hosmer and Lemeshow, 2000). We, therefore, ran 72 models (four domains * three sources of information * six discriminations). Discriminations of interest were structured in a hierarchical manner, comparing clinical groups with more impairment to groups with no or less impairment. Specifically, we tested the discrimination between Aβ− healthy controls and each of the other clinical groups among Aβ+ subjects (CN, MCI, AD), between Aβ+/CN, and each of the more impaired clinical groups (MCI, AD), and between MCI and AD. AUCs were adjusted for age, sex, and education level. The higher the AUC, the better the predictor is at distinguishing between two clinical groups. For each analysis, the specificity corresponding to a sensitivity of 80% was reported as the optimal cut-off score for that same sensitivity.

Finally, we tested whether there were significant differences in the accuracy of the three information sources in each of the six discrimination tasks mentioned above. We used the DeLong et al. (1988) method to perform pairwise comparisons between the accuracy (i.e., the AUCs) of the self-reported complaint, informant-reported complaint, and ACD. We used global ECog and ACD scores (and not by cognitive domain) to make the results more interpretable.

## Results

### Study Population

We included 380 Aβ+ subjects, distributed as follows: 31% had normal cognition (Aβ+/CN, *n* = 118), 50.3% had MCI (Aβ+/MCI, *n* = 191), and 18.7% had received a diagnosis of AD (Aβ+/AD, *n* = 71). We also included 211 Aβ−/CN subjects with normal cognition as healthy controls (Table 1).

**Table 1:** Baseline characteristics of the participants.

	Aβ-/CN	Aβ+ (*n* = 380)			*p*
	(controls)^a^	CN^b^	MCI^c^	AD^d^
	(*n* = 211)	(*n* = 118)	(*n* = 191)	(*n* = 71)
Age [years]	70.92 ± 5.89 (55.8–89)^b,c,d^	73.34 ± 6.46 (56.5–90.1)^a^	72.91 ± 6.92 (55-87.8)^a^	74.61 ± 7.83 (55.6–90.3)^a^	<0.01*
Education [years]	16.85 ± 2.41 (12–20)^d^	16.48 ± 2.64 (8-20)	16.22 ± 2.79 (9-20)	15.49 ± 2.46 (10-20)^a^	<0.01*
Sex [female]	106 (50.24%)^b^	84 (71.19%) ^a,c,d^	86 (45.03%)^b^	31 (43.66%)^b^	<0.01*
APOE-ε4 carriers	45 (21.84%)^b,c,d^	57 (51.90%)^a,c,d^	125 (67.02%)^a,b^	53 (77.94%)^a,b^	<0.01*
MMSE	29.16 ± 1.16 (24–30)^c,d^	28.97 ± 1.07 (26-30)^c,d^	27.89 ± 1.84 (19-30)^a,b,d^	22.73 ± 2.31 (18-26)^a,b,c^	<0.01*
Memory Score	1.1 ± 0.6 (−1.1 to 3.1)^c,d^	0.98 ± 0.56 (−0.7 to 2.7)^c,d^	0.25 ± 0.64 (−1.5 to 2.2)^a,b,d^	−0.92 ± 0.56 (−2.8-0.6)^a,b,c^	<0.01*
Language Score	0.24 ± 0.61 (−1.7 to 0.7)^d^	0.19 ± 0.57 (−1.5-0.7)^d^	−0.04 ± 0.73 (−2.5 to 0.7)^d^	−0.51 ± 0.95 (−3.2 to 0.7)^a,b,c^	<0.01*
Visuospatial Score	0.97 ± 0.71 (<0.9–3.1)^ d^	0.76 ± 0.69 (−1.2 to 2.8)^ d^	0.29 ± 0.79 (−1.9 to 2.6)^ d^	−0.78 ± 0.92 (−3.7 to 1.6)^a,b,c^	<0.01*
Executive Score	1.05 ± 0.80 (−1.2 to 3)^c,d^	0.78 ± 0.71 (<0.7 to 3)^c,d^	0.32 ± 0.92 (−1.9 to 3)^a,b,d^	−0.89 ± 0.93 (−3 to 1)^a,b,c^	<0.01*

Note. Results are given as mean ± standard deviation (Min-Max) or as n (%). APOE, Apolipoprotein; MMSE, Mini Mental State Examination; ECog, Everyday Cognition questionnaire. For the APOE genotype, the *n* and % represent the number and percentage of subjects presenting at least one ɛ4 allele. *Indicates statistical significance. Superscripted a, b, c, and d indicate significant pairwise between-group comparisons.

Aβ−/CN controls were younger than the other groups (*F*_(3,587)_ = 7.376, *η*^2^ = 0.036, *p* < 0.001) and had higher levels of education than Aβ+/AD subjects (*F*_(3,587)_ = 5.392, *η*^2^ = 0.027, *p* = 0.001). Women were overrepresented in the Aβ+/CN group (about 71%, χ^2^ = 23.632, *p* < 0.001 compared to men). The number of APOE ε4 carriers differed between groups, *F*_(3,568)_ = 42.790, *η*^2^ = 0.189, *p* < 0.001 (Aβ−/CN < Aβ+/CN < Aβ+/MCI < Aβ+/AD, the latter difference not being statistically significant). All further analyses were controlled for age, sex and education.

The Memory and Executive composites were significantly different between groups (Aβ−/CN = Aβ+/CN > Aβ+/MCI > Aβ+/AD; Memory: *η*^2^ = 0.51, *p* < 0.01; Executive: *η*^2^ = 0.28, *p* < 0.01). The Language and Visuospatial composite scores were on average significantly lower (indicating greater impairment) in the AD group than in the other groups (Language: *η*^2^ = 0.29, *p* < 0.01; Visuospatial: *η*^2^ = 0.08, *p* < 0.01).

#### Objective 1: Comparisons of ECog-Subject, ECog-StudyPartner, and ACD by Cognitive Domain and Clinical Group

**Figure 1:**
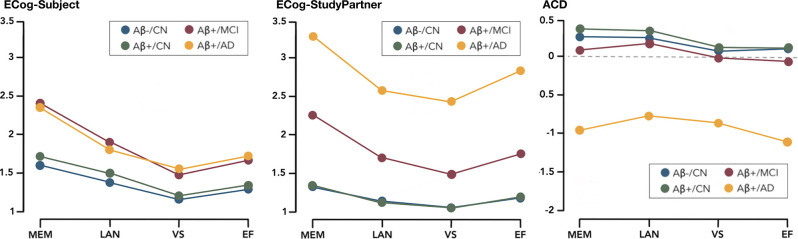
ECog-Subject, ECog-StudyPartner, and ACD by domain between groups.

Figure 1 and Table 2 show the patterns of cognitive complaints (ECog-Subject, ECog-StudyPartner) and ACD across the four investigated domains (Memory, Language, Visuospatial abilities, and Executive functions) in the four groups (CN/Aβ+, MCI/Aβ+, AD/Aβ+, and CN/Aβ−). The analyses for Objective 1 were controlled for age, sex, and education. Detailed statistical indices for Objective 1 are available in Supplementary Materials.

**Table 2:** Comparison of ECog-Subject, ECog-StudyPartner, and ACD by Domain between Groups.

SELF-REPORTED COMPLAINT (ECog-Subject)						
Memory	Language	Visuospatial abilities	Executive functions	*p*	Intragroup effects
Aβ-/CN	1.60 ± 0.52	1.38 ± 0.40	1.15 ± 0.27	1.28 ± 0.34	<0.01	M > L > E > V
Aβ+/CN	1.71 ± 0.47	1.49 ± 0.43	1.18 ± 0.26	1.32 ± 0.32	<0.01	M > L > E > V
Aβ+/MCI	2.38 ± 0.70	1.90 ± 0.68	1.48 ± 0.59	1.67 ± 0.61	<0.01	M > L > E > V
Aβ+/AD	2.34 ± 0.78	1.80 ± 0.67	1.56 ± 0.58	1.71 ± 0.61	<0.01	M > L = E > V
*p*	<0.01	<0.01	<0.01	<0.01		
Intergroup effects	Aβ-/CN = Aβ+/C < MCI = AD	Aβ-/CN = Aβ+/C < MCI = AD	Aβ-/CN = Aβ+/C < MCI = AD	Aβ-/CN = Aβ+/CN < MCI = AD		
INFORMANT-REPORTED COMPLAINT (ECog-StudyPartner)						
	Memory	Language	Visuospatial abilities	Executive functions	*p*	Intragroup effects
Aβ-/CN	1.32 ± 0.43	1.13 ± 0.24	1.06 ± 0.16	1.17 ± 0.34	<0.01	M > E = L > V
Aβ+/CN	1.33 ± 0.43	1.12 ± 0.22	1.06 ± 0.15	1.18 ± 0.33	<0.01	M > E = L = V
Aβ+/MCI	2.27 ± 0.83	1.70 ± 0.69	1.48 ± 0.61	1.73 ± 0.70	<0.01	M > E = L > V
Aβ+/AD	3.28 ± 0.63	2.57 ± 0.76	2.41 ± 0.84	2.81 ± 0.76	<0.01	M > E = L = V
*p*	<0.01	<0.01	<0.01	<0.01		
Intergroup effects	Aβ-/CN = Aβ+/CN < MCI < AD	Aβ-/CN = Aβ+/CN < MCI < AD	Aβ-/CN = Aβ+/CN < MCI < AD	Aβ-/CN = Aβ+/CN < MCI < AD	
AWARENESS OF COGNITIVE DECLINE, ACD (ECog-Subject *minus* ECog-StudyPartner)						
	Memory	Language	Visuospatial abilities	Executive functions	*p*	Intragroup effects
Aβ-/CN	0.28 ± 0.55	0.25 ± 0.42	0.09 ± 0.27	0.11 ± 0.36	<0.01	M = L > E = V
Aβ+/CN	0.38 ± 0.47	0.36 ± 0.41	0.12 ± 0.26	0.13 ± 0.37	<0.01	M = L > E = V
Aβ+/MCI	0.10 ± 0.89	0.20 ± 0.88	0.00 ± 0.78	<0.06 ± 0.83	<0.01	L > E = V; M > E; L = M; M =V
Aβ+/AD	<0.94 ± 1.00	<0.77 ± 0.88	<0.85 ± 0.95	−1.10 ± 0.90	0.01	M = L, E, V; L > E; V > E
*p*	<0.01	<0.01	<0.01	<0.01		
Intergroup effects	Aβ-/CN = Aβ+/CN = MCI > AD	Aβ-/CN = Aβ+/CN = MCI > AD	Aβ-/CN = Aβ+/CN = MCI > AD	Aβ-/CN = Aβ+/CN = MCI > AD		

*Note. Results are given as mean ± standard deviation. In the intragroup effects, M, Memory; L, Language; V, Visuospatial abilities; E, Executive functions. In the intergroup and intragroup effects, > indicates “significantly higher than”; <indicates “significantly lower than”, = indicates “not significantly different”*.

### ECog-Subject Scores

The effect of the Group*Domain interaction was significant (*F*_(91,761)_ = 16.761, partial *η*^2^ = 0.016, *p* < 0.001; Table 2).

In all groups combined, the ECog-Subject scores were significantly different in each cognitive domain(*F*_(31,761)_ = 422.787, partial *η*^2^ = 0.131, *p* < 0.001). *Post-hoc* pairwise comparison showed that memory was globally the domain in which the participants reported the greatest complaints, followed by language, executive functions, and finally, visuospatial abilities. The only exception was in Aβ+/AD participants, in which language and executive function complaints were not significantly different.

The ECog-Subject scores also differed significantly between the groups (*F*_(3,587)_ = 55.175, partial *η*^2^ = 0.220, *p* < 0.001). Aβ+/CN participants and Aβ-/CN controls reported complaints of similar intensity, while Aβ+/MCI and Aβ+/AD participants reported significantly greater difficulties than the two groups of CN participants. No significant difference was observed between Aβ+/MCI and Aβ+/AD participants.

### ECog-StudyPartner Scores

The effect of the Group*Domain interaction was significant (*F*_(91,761)_ = 28.476, partial *η*^2^ = 0.018, *p* < 0.001).

In all groups combined, the ECog-StudyPartner scores were significantly different in each cognitive domain (*F*_(31,761)_ = 270.578, partial *η*^2^ = 0.057, *p* < 0.001). More specifically, the study-partners reported that memory was the most impaired cognitive domain in the subjects, followed by language and executive functions, with no differences between these two. Complaints regarding visuospatial abilities were significantly less intense than in the other domains in Aβ-/CN and Aβ+/MCI, but not in Aβ+/CN and Aβ+/AD.

The ECog-StudyPartner score also differed significantly between the groups (*F*_(3,587)_ = 262.240, partial *η*^2^ = 0.573, *p* < 0.001). *Post-hoc* pairwise comparisons showed that study-partners of Aβ+/CN subjects and Aβ-/CN controls globally reported complaints of similar intensity, followed by—in increasing order—Aβ+/MCI and Aβ+/AD.

### Awareness of Cognitive Decline

The effect of the Group*Domain interaction was significant (*F*_(91,761)_ = 28.476, partial *η*^2^ = 0.018, *p* < 0.001).

In all groups combined, the ACD scores were significantly different in each cognitive domain(*F*_(31,761)_ = 42.301, partial *η*^2^ = 0.013, *p* < 0.001). In both Aβ-/CN and Aβ+/CN, awareness of memory and language performance was higher than awareness of visuospatial and executive performance. In Aβ+/MCI, awareness of memory and language performance was significantly higher than awareness of executive function, and awareness of language performance was higher than awareness of visuospatial performance. Finally, in Aβ+/AD, awareness of executive performance was significantly poorer than awareness for visuospatial and language performance.

The ACD also differed significantly between the groups (*F*_(3,587)_ = 75.646, partial *η*^2^ = 0.279, *p* < 0.001). *Post-hoc* pairwise comparisons showed that Aβ+/AD participants had significantly lower ACD than all other groups, regardless of the cognitive domain.

#### Objective 2: Discriminant Value of Ecog-Subject, Ecog-StudyPartner, and Awareness of Cognitive Decline Per Cognitive Domain

Table 3 summarizes the ROC curve analysis with specificity (at 80% of sensitivity) for each diagnostic comparison. It shows how accurately the ECog and ACD scores in each cognitive domain can discriminate clinical groups (six pairwise discriminations between Aβ-/CN, Aβ+/CN, Aβ+/MCI, and Aβ+/AD groups).

**Table 3:** Results of ROC/AUC analysis.

	ECog-Subject		ECog-Study Partner		ACD	
	AUC	Specificity at sensitivity = 0.8	AUC	Specificity at sensitivity = 0.8	AUC	Specificity at sensitivity = 0.8
Memory					
Aβ+/CN vs. Aβ</CN	0.70	0.31	0.69	0.22	0.70	0.20
Aβ+/MCI vs. Aβ-/CN	**0.83**	0.69	**0.86**	0.74	0.63	0.15
Aβ+/AD vs. Aβ-/CN	**0.85**	0.59	**0.98**	0.99	**0.88**	0.73
Aβ+/MCI vs. Aβ+/CN	0.78	0.57	**0.87**	0.79	0.71	0.15
Aβ+/AD vs. Aβ+/MCI	0.60	0.14	**0.83**	0.73	0.77	0.52
Aβ+/AD vs. Aβ+/CN	**0.80**	0.46	**0.99**	0.99	**0.93**	0.76
Language						
Aβ+/CN vs. Aβ-/CN	0.70	0.30	0.70	0.21	0.70	0.27
Aβ+/MCI vs. Aβ-/CN	0.75	0.54	**0.81**	0.63	0.61	0.03
Aβ+/AD vs. Aβ-/CN	0.78	0.42	**0.97**	0.98	**0.86**	0.61
Aβ+/MCI vs. Aβ+/CN	0.75	0.41	**0.83**	0.63	0.69	0.14
Aβ+/AD vs. Aβ+/MCI	0.61	0.17	**0.80**	0.67	0.78	0.57
Aβ+/AD vs. Aβ+/CN	0.75	0.30	**0.98**	0.98	**0.91**	0.72
Visuospatial Ability						
Aβ+/CN vs. Aβ-/CN	0.70	0.25	0.70	0.22	0.70	0.22
Aβ+/MCI vs. Aβ-/CN	0.73	0.30	0.79	0.36	0.62	0.08
Aβ+/AD vs. Aβ-/CN	**0.81**	0.58	**0.96**	0.97	**0.84**	0.92
Aβ+/MCI vs. Aβ+/CN	0.75	0.24	**0.82**	0.32	0.79	0.09
Aβ+/AD vs. Aβ+/MCI	0.61	0.29	**0.82**	0.66	**0.85**	0.64
Aβ+/AD vs. Aβ+/CN	**0.80**	0.52	**0.96**	0.98	**0.88**	0.93
Executive Functions						
Aβ+/CN vs. Aβ-/CN	0.70	0.30	0.70	0.20	0.70	0.28
Aβ+/MCI vs. Aβ-/CN	0.73	0.52	**0.80**	0.64	0.64	0.11
Aβ+/AD vs. Aβ-/CN	**0.81**	0.52	**0.97**	0.98	**0.91**	0.92
Aβ+/MCI vs. Aβ+/CN	0.74	0.44	**0.81**	0.66	0.69	0.14
Aβ+/AD vs. Aβ+/MCI	0.61	0.20	**0.85**	0.74	**0.82**	0.68
Aβ+/AD vs. Aβ+/CN	0.79	0.44	**0.97**	0.95	**0.91**	0.90

*Note. AUC, Area Under the ROC; ACD, Awareness of Cognitive Decline. To facilitate understanding of the table, all AUCs between 0.80 and 0.90 are in bold, and all AUCs > 0.90 are in bold and underlined*.

### Discriminant Value of ECog-Subject by Domain

ECog-Subject scores performed globally better than chance in distinguishing between groups, although they did not have excellent accuracy: the highest AUC was 0.85, AUC above 0.80 was not frequent and specificities were inferior to 69%.

The best performance of the ECog-Subject was in the discrimination between MCI and Aβ-/CN (AUC memory = 0.83), between AD and Aβ-/CN (AUC memory = 0.85, AUC executive function = 0.81, AUC visuospatial = 0.81) and between AD and Aβ+/CN (AUC memory = 0.80, AUC visuospatial = 0.80).

The worst performances were in the discrimination between the two CN groups (all AUCs = 0.70), and between MCI and AD (all AUCs between 0.60 and 0.61).

### Discriminant Value of ECog-StudyPartner by Domain

ECog-StudyPartner scores showed good to excellent accuracy in almost all discriminations. The best performance of the Ecog-StudyPartner scores was in the discrimination between AD and Aβ-/CN (AUCs between 0.96 and 0.98) and between AD and Aβ+/CN (AUCs between 0.96 and 0.99). Specificities could reach very high levels (99% as a maximum).

No ECog-StudyPartner score (i.e., in any cognitive domain) seems useful to distinguish Aβ+/CN and Aβ-/CN subjects (all AUCs between 0.69 and 0.70).

### Discriminant Value of Awareness of Cognitive Decline by Domain

ACD scores showed good to excellent accuracy in the discrimination between AD and Aβ+/CN (AUCs between 0.88 and 0.93), between AD and Aβ-/CN (AUCs between 0.84 and 0.91), and between AD and MCI (AUC_ef_ = 0.82, AUC_vs_ = 0.85). In these discriminations, specificities could reach very high levels (99% as a maximum), especially in the visuospatial and executive domains. Accuracies were low to moderate in the other discriminations (AUCs between 0.61 and 0.79).

### Comparison of the Three Sources of Information

Globally, the ECog-StudyPartner performed significantly better than the other two sources of information in all discriminations, except Aβ−/CN vs. Aβ+/CN, where the three sources of information did not differ significantly (all AUCs between 0.69 and 0.70); ECog-Subject vs. ECog-StudyPartner: *Z* = −1.65, *p* = 0.09; ECog-StudyPartner vs. ACD: *Z* = −1.89, *p* = 0.06; ECog-Subject vs. ACD: *Z* = −0.54, *p* = 0.59.

**Figure 2:**
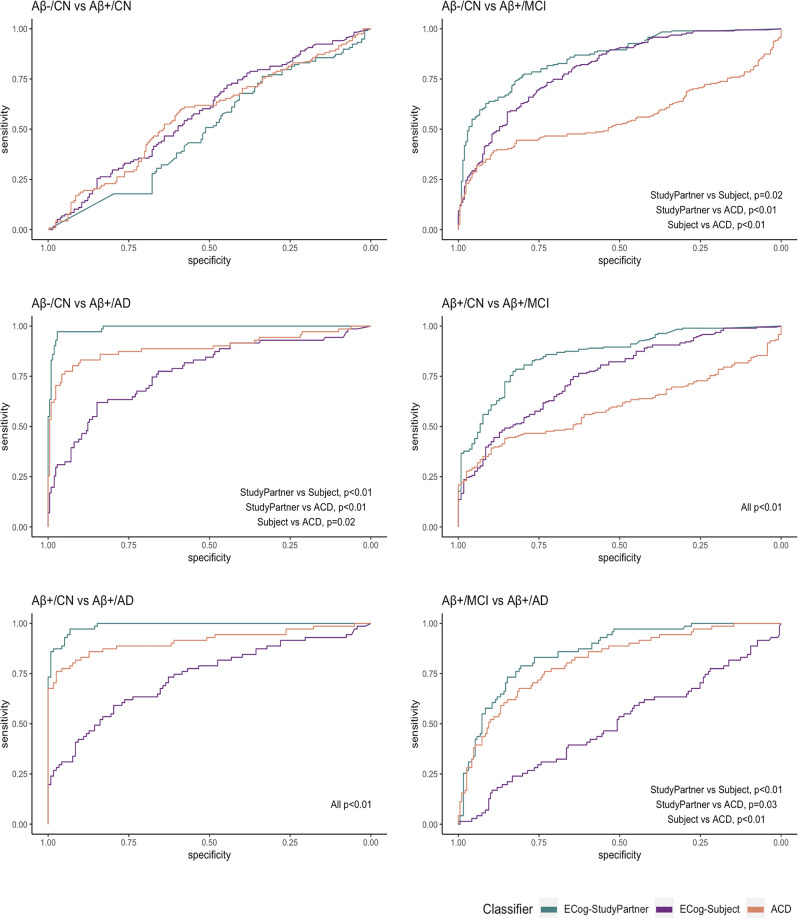
ROC comparisons between the three sources of information.

ACD was significantly less accurate than ECog-Subject in two out of six discriminations, namely Aβ−/CN vs. MCI (ECog-Subject vs. ACD: *Z* = 5.47, *p* <0.01) and Aβ+/CN vs. MCI (ECog-Subject vs. ACD: *Z* = 3.01, *p* <0.01). The ACD score was significantly more accurate than the ECog-Subject in the other discriminations. More details are in Figure 2.

### *Post-hoc* Analysis: Correlation Between Subjective and Objective Cognitive Measures

The results from the analysis comparing the various sources of information suggest that the ECog-StudyPartner performs better at discriminating the groups than the ECog-Subject. One possible interpretation is that informants are more accurate at assessing the cognitive levels of the subjects than the subjects themselves. To explore this interpretation, we carried Pearson’s correlations between objective measures of cognition (four composite scores) and subjective measures (four ECog-Subject, four ECog-StudyPartner). To compare the correlation coefficients obtained between the objective measures of cognition and the ECog-Subject, versus the correlation coefficients obtained between the objective measures and the ECog-StudyPartner, we performed tests of significance using the “cocor” R package for the comparison of two overlapping correlations based on dependent groups.

In both subjects and informants, the cognitive composite scores correlated significantly and negatively with the cognitive complaints in all four cognitive domains (Table 4). The negative correlations indicate that elevated cognitive complaints are associated with lower objective cognitive performance. When comparing the correlation coefficients between subjects and informants, we found that the objective composite scores were significantly more strongly correlated with the ECog-StudyPartner (all *r* between −0.29 and −0.64) than with the ECog-Subject (all *r* between −0.16 and −0.38), for all cognitive domains. A more extensive correlation matrix is also included in the Supplementary Materials.

**Table 4:** Comparison of correlation coefficients between subjective and objective measures of cognitive decline.

	Same-domain	Same-domain	*p*-values
	ECog-Subject	ECog StudyPartner	
Memory composite	−0.38*	−0.64*	<0.01
Language composite	−0.34*	−0.51*	<0.01
Visuospatial composite	−0.32*	−0.49*	<0.01
Executive composite	−0.16*	−0.29*	<0.01

Note. The table reports correlation coefficients between each of the four composite scores and the same-domain ECog-Subject and ECog-StudyPartner, separately. *Indicates significant correlations. *p*-values refer to the pairwise comparisons between correlation coefficients.

## Discussion

In this study, we investigated domain-specific cognitive complaints across the amnestic AD spectrum (more precisely, in amyloid-positive individuals ranging from normal cognition to dementia) and controls, using three sources of information: self-reported complaints, informant-reported complaints, and the discrepancy between these two reports as a measure of awareness of cognitive decline (ACD). To briefly recap the main findings of this study, the intensity of cognitive complaints, both self- and informant-reported, was generally distributed according to the following trend: memory, language, executive, and visuospatial (from most to least impaired). The two groups with normal cognition (i.e., amyloid negative and positive) reported experiencing a more marked decline in memory and language than noticed by their informants. The Aβ+/MCI participants had good agreement with their informants, while AD participants presented poor ACD (anosognosia), especially for the executive domain. In terms of the ability of these sources of information to discriminate between groups, we found that informant-reported cognitive complaints in all domains performed the best. ACD scores, in all domains, accurately distinguish AD from CN participants. Self-reported complaints were not as accurate in discriminating the groups. Finally, while both self-reported and informant-reported complaints were correlated with objective cognitive scores in each cognitive domain, informant-reported complaints were significantly more correlated with objective cognitive scores than self-reported complaints.

### Amnestic and Non-amnestic Cognitive Complaints Across the Amnestic AD Spectrum

Subjects and study-partners from all groups reported the most complaints in the memory domain. This was expected given the inclusion criteria of ADNI, which requires significant memory complaints in the MCI and AD participants. Episodic memory is also the most frequently impaired cognitive domain in AD (Sarazin et al., 2007). Language and executive functions were the domains reported to be most impaired after memory. Language and executive disorders appear quite early in the course of the disease and become more and more marked in the patient’s clinical picture (Ahmed et al., 2013; Harrington et al., 2013). For instance, a recent study including healthy controls, cognitive-complainers without objective deficit (hence with *subjective cognitive decline* or SCD, Jessen et al., 2020), and patients with AD found that the majority of subjects reported memory complaints (including 26% of healthy controls) but also language complaints (including 37% of controls; Miebach et al., 2019). Finally, subjects and study-partners from all groups reported visuospatial disorders to be the least intense compared to the other domains. Indeed, visuospatial disorders, such as difficulty in the spatial localization of objects, generation of mental pathways, and spatial navigation, might occur later in the course of the disease (Cherrier et al., 2001). In our study, Aβ+/CN and MCI subjects performed similarly to healthy controls on the visuospatial composite score, while only AD patients performed significantly worse. Recent studies show that mild visuospatial disorders may also be present in early-stage AD (Joray et al., 2004), but it must be noted that these are difficulties that the patient and those around them may not recognize in daily life until that they become more severe.

### The Clinical Utility of Domain-Specific Informant-Reported Cognitive Complaints

Informant-reported cognitive complaints were globally the best measures to distinguish groups, in comparison to self-reported complaints and ACD, consistent with what has already been identified from previous studies (Gifford, 2015; Rueda, 2015). Therefore, this source of information should be prioritized by clinicians during clinical interviews. All ECog-StudyPartner scores were good to excellent predictors for discriminating groups of individuals at different stages of AD and controls. The only discrimination in which the informant report was not sufficiently sensitive was between Aβ+/CN from Aβ-/CN participants. This suggests that the informant report, as measured by the ECog, is not sensitive enough to detect the disease when the patient is asymptomatic.

Furthermore, informant reports strongly correlated with the same-domain composite cognitive scores, suggesting that they may be taken as a gold standard to collect information about the patient’s cognitive functioning in daily life. Nonetheless, we acknowledge that informant-reported complaints may be potentially biased by factors such as anxiety, depression, caregiver burden, or personality traits. However, study-partners were accurate in previous studies despite these potential biases: in a study by Cacchione and colleagues, the accuracy of the study-partner in predicting patient’s cognitive decline was above chance even for informants who were not spouses, who did not live with the patient, or who spoke with the patient less than daily, and for older or less educated patients (Cacchione et al., 2003).

### Self-Reported Cognitive Complaints and Subject’s Self-Awareness

In the present study, the self-reported complaints were the less accurate measures to distinguish groups along the AD spectrum. Although our *post-hoc* analysis showed that self-reported complaints correlated significantly with objective cognitive scores, the strength of associations was significantly weaker than between informant-reported complaints and objective cognitive scores. Also, in our sample, some individuals tended to underestimate their cognitive abilities (especially CN subjects), while others overestimated them (especially AD subjects). On the other hand, the ACD measure was slightly more accurate than self-report complaints but less accurate than the informant-report. It would be interesting to understand if the subject-informant discrepancy can better discriminate patients with different pathologies than the informant-report alone. Although progressive anosognosia is a common symptom of several neurological or psychiatric diseases—e.g., frontotemporal dementia (Zamboni et al., 2010) or Huntington’s disease (Hoth, 2007), identifying a certain degree of anosognosia could be useful in the differential diagnosis.

Aβ+/CN subjects (at risk for preclinical AD) and Aβ−/CN controls reported complaints of similar intensity, and this measure discriminated the two CN groups slightly better than chance. When relating the self-reported complaint to the informant-reported complaint (ACD score) we found that both CN groups exhibited more marked memory and language complaints than their informants, and this was not the case with executive and visuospatial complaints. This is consistent with a previous study highlighting the importance of word-finding complaints in CN, on top of memory complaints (Montembeault et al., 2022). The difference between self- and informant-reported complaints in CN may be consistent with the concept of *hypernosognosia* (Vannini et al., 2017), a term used when cognitively unimpaired individuals with high levels of amyloid deposition perceive a subtle decline in memory and language that their informant does not notice yet. On the other hand, since this pattern (memory and language ECog-Subject >StudyPartner) was also observed in control subjects, it may suggest that cognitive complaints are nonspecific and common even among healthy individuals. Previous studies also found that most healthy elderly express some degree of cognitive complaints (Jessen, 2010; van Harten, 2018). This may be partially related to anxiety, depression, medication intake, and age-related cognitive changes (Buckley, 2013). Nonetheless, many studies have demonstrated a relationship between cognitive complaints and amyloid status (La Joie et al., 2016; Valech et al., 2018; Miebach et al., 2019; Montembeault et al., 2022). Another noteworthy aspect to discuss is that our control subjects were not from the general population but were part of a cohort selected to study AD, presenting with memory complaints at inclusion, which could have affected the results.

MCI participants and their study-partner reported similar levels of cognitive decline across all domains, meaning they did not show anosognosia. In some previous studies, MCI patients exhibited marked cognitive complaints (more marked than informant-reported complaints; Kalbe et al., 2005; Piras et al., 2016), while others found mild anosognosia (e.g., Hanseeuw et al., 2020; Cacciamani et al., 2021). These conflicting findings on self-awareness in MCI are likely due to the heterogeneity of the concept of MCI itself, in addition to a known inter-individual variability in the rate of disease progression and in the ordering of symptom onset (Goyal et al., 2018).

Concerning AD participants, they did not perceive more cognitive impairment than those with MCI despite the fact that they had more marked disorders at testing, which were also noticed by their study-partner. Indeed, AD participants presented with anosognosia. These results are consistent with the *petrified self theory*, suggesting that anosognosia in AD may be due to patients’ self-assessment being petrified or anchored to their pre-morbid abilities (Mograbi et al., 2009). They may recognize their cognitive errors soon after they are made, but the knowledge about these failures is only partially and temporarily incorporated into their self-knowledge (Mograbi et al., 2009; Kalenzaga and Clarys, 2013). Thus, the subjective perception of decline would not coincide with the actual progression of cognitive impairment. The ACD measure that performed best on the AUC analysis was the Executive.

Function subscale. This means that anosognosia for executive function disorders is the most sensitive measure to distinguish individuals with dementia from other groups, among the four domains considered. Another study has shown that the level of ACD differs depending on the object studied in AD patients, with awareness of the overall condition and executive functions and for the overall condition being the most impaired, while the awareness of disinhibition and apathy was more preserved (Bertrand et al., 2019). This reiterates that the investigation of domains other than episodic memory could provide added value of clinical utility.

## Limitations

This study has some limitations. The main limitation of this study is that the diagnosis of MCI and AD in the ADNI cohort is partly made on the basis of memory complaints. Although the main variables of interest in the present study are also cognitive complaints which could lead to circularity, it is important to note that the complaints used for diagnosis were strictly amnestic (did not concern other cognitive domains) and were reported during the clinical interview (not measured using the ECog). Nonetheless, this had an impact on our results. First, because cognitive complaints were required for inclusion in the MCI and AD group, but not for the two CN groups, it was expected that complaints would be more elevated in MCI and AD versus CNs. However, this limitation does not affect the comparison of cognitive complaints in MCI vs. AD and in Aβ−/CN vs. Aβ+/CN. Secondly, because our MCI and AD population were amnestic, it was expected that cognitive complaints would be more elevated in the memory domain than in other cognitive domains. Nonetheless, the current study provides novel knowledge on non-memory cognitive complaints in this population. To verify the generalisability of our results, a population-based cohort with no criteria for memory complaints could be studied. A second limitation is that we have no information about the study-partner, for example, the degree of kinship with the subject, how long they have known the subject, and how much time they spend with them. However, the strong correlation with cognitive score suggests that informant-related complaints are representative of objective cognitive measures. Finally, it was not possible to use the level of tau protein as it was not available in many subjects. This may have led to a bias in the selection of subjects. Indeed, it would have been more precise if it were based on the two biomarkers, amyloid, and tau (Jack et al., 2018).

## Concluding Remarks

Our results can have interesting applications for both research and clinical practice. They highlight the limitations and benefits of three sources of information that are valuable to the clinician and the researcher, namely self-reported complaint, informant-reported complaint, and concordance or discrepancy between the two (as a measure of ACD), all relating to different cognitive domains. The inclusion of an informant or study-partner seems to be an important added value for an accurate, early diagnosis, and for effective selection of individuals in clinical trials. Given the predictive power of study-partner complaint in disease staging, further studies could identify thresholds of abnormality of the ECog-StudyPartner score for use in clinical practice. The patients themselves, on the contrary, are less accurate in their reports and may tend to both overestimate their abnormal performance (as a form of anosognosia) or underestimate their normal performance (as in worried-well individuals). These results also suggest that patients and study-partners complain not only about memory but also about other cognitive domains, and non-amnesic complaints and ACD also provided important clinical information. This is important to emphasize, as the current research criteria defining complaints typical of AD patients are memory-related (Jessen et al., 2020), and we believe they should be revised to also include non-amnesic complaints. To facilitate the application of these results in clinical practice, an interesting perspective for future studies is to understand whether there are specific questions, relating to the different cognitive domains, to ask the subject and the informant in order to detect the disease earlier. Much attention has been paid to memory complaints (e.g., Jessen et al., 2020) and awareness of memory disorders (e.g., Gagliardi et al., 2020), but the clinical presentation of AD is more diverse (Goyal et al., 2018). Focusing solely on memory could, for example, exclude all patients with a non-amnestic phenotype.

## Data Availability Statement

The dataset is owned by the Alzheimer’s Disease Neuroimaging Initiative (ADNI). Data are publicly and freely available from http://adni.loni.usc.edu upon sending a request.

## Ethics Statement

The studies involving human participants were reviewed and approved by Good Clinical Practice guidelines, US 21CFR Part 50—Protection of Human Subjects, and Part 56—Institutional Review Boards (IRBs)/Research Ethics Boards (REBs), and state and federal HIPAA regulations. The patients/participants provided their written informed consent to participate in this study.

## Author Contributions

All authors contributed to the study design and provided expertise and insights into the interpretation of the results. FC, VG, and MM provided statistical expertise. The manuscript was drafted by FC, VG, and MM, and critically reviewed and approved by all authors. All authors contributed to the article and approved the submitted version.

## Conflict of Interest

SE has received honoraria as a speaker or consultant for Eli Lilly, Biogen, Astellas Pharma, Roche, and GE Healthcare. The remaining authors declare that the research was conducted in the absence of any commercial or financial relationships that could be construed as a potential conflict of interest.

## Publisher’s Note

All claims expressed in this article are solely those of the authors and do not necessarily represent those of their affiliated organizations, or those of the publisher, the editors and the reviewers. Any product that may be evaluated in this article, or claim that may be made by its manufacturer, is not guaranteed or endorsed by the publisher.
