# Supplementary material for: Differential Patterns of Domain-Specific Cognitive Complaints and Awareness Across the Alzheimer’s Disease Spectrum
Source: Front Aging Neurosci. 2022 Jun 16;14:811739. doi: 10.3389/fnagi.2022.811739 (PMC9261482; doi:10.3389/fnagi.2022.811739)
Supplement: Supplementary file 1 [file Table_1.docx]

**Supplementary material**

*Figure S1. Main effect of* Domain *on ECog-Subject, ECog-StudyPartner and ACD*


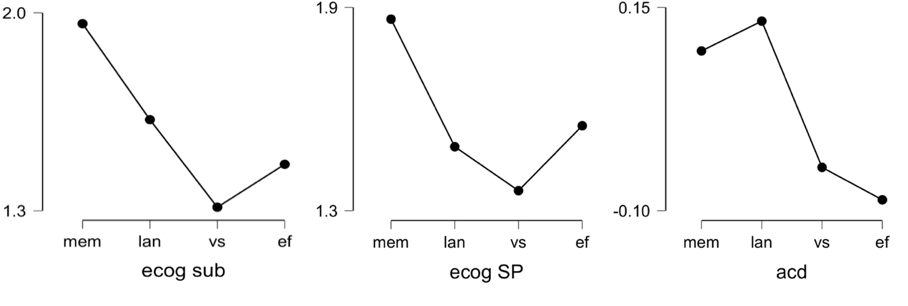


*Figure S2. Main effect of* Group *on ECog-Subject, ECog-StudyPartner and ACD*


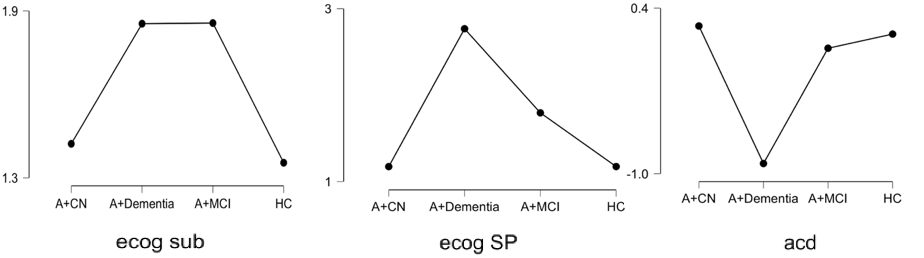


| Table S1. Post Hoc Comparisons – ECog-Subject ✻ groupe | | | | | |
| --- | --- | --- | --- | --- | --- |
|  |  | Mean Difference | SE | t | p _bonf_ |
| ef,A+CN | ef,A+AD | -0.394 | 0.078 | -5.025 | < .001 |
|  | ef,A+MCI | -0.350 | 0.061 | -5.715 | < .001 |
|  | ef,HC | 0.035 | 0.060 | 0.575 | 1.000 |
|  | lan,A+CN | -0.169 | 0.040 | -4.234 | 0.003 |
|  | lan,A+AD | -0.488 | 0.078 | -6.214 | < .001 |
|  | lan,A+MCI | -0.588 | 0.061 | -9.605 | < .001 |
|  | lan,HC | -0.066 | 0.060 | -1.096 | 1.000 |
|  | mem,A+CN | -0.391 | 0.040 | -9.777 | < .001 |
|  | mem,A+AD | -1.028 | 0.078 | -13.091 | < .001 |
|  | mem,A+MCI | -1.056 | 0.061 | -17.259 | < .001 |
|  | mem,HC | -0.287 | 0.060 | -4.784 | < .001 |
|  | vs,A+CN | 0.135 | 0.040 | 3.384 | 0.088 |
|  | vs,A+AD | -0.241 | 0.078 | -3.072 | 0.262 |
|  | vs,A+MCI | -0.166 | 0.061 | -2.717 | 0.805 |
|  | vs,HC | 0.166 | 0.060 | 2.768 | 0.689 |
| ef,A+AD | ef,A+MCI | 0.045 | 0.073 | 0.616 | 1.000 |
|  | ef,HC | 0.429 | 0.072 | 5.983 | < .001 |
|  | lan,A+CN | 0.225 | 0.078 | 2.870 | 0.502 |
|  | lan,A+AD | -0.093 | 0.052 | -1.811 | 1.000 |
|  | lan,A+MCI | -0.193 | 0.073 | -2.661 | 0.949 |
|  | lan,HC | 0.329 | 0.072 | 4.583 | < .001 |
|  | mem,A+CN | 0.004 | 0.078 | 0.048 | 1.000 |
|  | mem,A+AD | -0.633 | 0.052 | -12.291 | < .001 |
|  | mem,A+MCI | -0.662 | 0.073 | -9.108 | < .001 |
|  | mem,HC | 0.107 | 0.072 | 1.493 | 1.000 |
|  | vs,A+CN | 0.530 | 0.078 | 6.748 | < .001 |
|  | vs,A+AD | 0.153 | 0.052 | 2.977 | 0.354 |
|  | vs,A+MCI | 0.228 | 0.073 | 3.142 | 0.207 |
|  | vs,HC | 0.561 | 0.072 | 7.821 | < .001 |
| ef,A+MCI | ef,HC | 0.384 | 0.052 | 7.362 | < .001 |
|  | lan,A+CN | 0.181 | 0.061 | 2.951 | 0.389 |
|  | lan,A+AD | -0.138 | 0.073 | -1.900 | 1.000 |
|  | lan,A+MCI | -0.238 | 0.031 | -7.580 | < .001 |
|  | lan,HC | 0.284 | 0.052 | 5.439 | < .001 |
|  | mem,A+CN | -0.041 | 0.061 | -0.669 | 1.000 |
|  | mem,A+AD | -0.678 | 0.073 | -9.331 | < .001 |
|  | mem,A+MCI | -0.706 | 0.031 | -22.492 | < .001 |
|  | mem,HC | 0.062 | 0.052 | 1.194 | 1.000 |
|  | vs,A+CN | 0.485 | 0.061 | 7.925 | < .001 |
|  | vs,A+AD | 0.109 | 0.073 | 1.495 | 1.000 |
|  | vs,A+MCI | 0.183 | 0.031 | 5.843 | < .001 |
|  | vs,HC | 0.516 | 0.052 | 9.887 | < .001 |
| ef,HC | lan,A+CN | -0.204 | 0.060 | -3.391 | 0.087 |
|  | lan,A+AD | -0.522 | 0.072 | -7.284 | < .001 |
|  | lan,A+MCI | -0.622 | 0.052 | -11.923 | < .001 |
|  | lan,HC | -0.100 | 0.030 | -3.360 | 0.095 |
|  | mem,A+CN | -0.425 | 0.060 | -7.078 | < .001 |
|  | mem,A+AD | -1.062 | 0.072 | -14.813 | < .001 |
|  | mem,A+MCI | -1.091 | 0.052 | -20.896 | < .001 |
|  | mem,HC | -0.322 | 0.030 | -10.774 | < .001 |
|  | vs,A+CN | 0.101 | 0.060 | 1.676 | 1.000 |
|  | vs,A+AD | -0.276 | 0.072 | -3.845 | 0.015 |
|  | vs,A+MCI | -0.201 | 0.052 | -3.847 | 0.015 |
|  | vs,HC | 0.132 | 0.030 | 4.409 | 0.001 |
| lan,A+CN | lan,A+AD | -0.319 | 0.078 | -4.059 | 0.006 |
|  | lan,A+MCI | -0.419 | 0.061 | -6.841 | < .001 |
|  | lan,HC | 0.103 | 0.060 | 1.720 | 1.000 |
|  | mem,A+CN | -0.222 | 0.040 | -5.544 | < .001 |
|  | mem,A+AD | -0.858 | 0.078 | -10.936 | < .001 |
|  | mem,A+MCI | -0.887 | 0.061 | -14.494 | < .001 |
|  | mem,HC | -0.118 | 0.060 | -1.968 | 1.000 |
|  | vs,A+CN | 0.304 | 0.040 | 7.617 | < .001 |
|  | vs,A+AD | -0.072 | 0.078 | -0.917 | 1.000 |
|  | vs,A+MCI | 0.003 | 0.061 | 0.048 | 1.000 |
|  | vs,HC | 0.335 | 0.060 | 5.584 | < .001 |
| lan,A+AD | lan,A+MCI | -0.100 | 0.073 | -1.377 | 1.000 |
|  | lan,HC | 0.422 | 0.072 | 5.884 | < .001 |
|  | mem,A+CN | 0.097 | 0.078 | 1.237 | 1.000 |
|  | mem,A+AD | -0.540 | 0.052 | -10.480 | < .001 |
|  | mem,A+MCI | -0.568 | 0.073 | -7.824 | < .001 |
|  | mem,HC | 0.200 | 0.072 | 2.794 | 0.636 |
|  | vs,A+CN | 0.623 | 0.078 | 7.936 | < .001 |
|  | vs,A+AD | 0.247 | 0.052 | 4.788 | < .001 |
|  | vs,A+MCI | 0.322 | 0.073 | 4.426 | 0.001 |
|  | vs,HC | 0.654 | 0.072 | 9.122 | < .001 |
| lan,A+MCI | lan,HC | 0.522 | 0.052 | 9.999 | < .001 |
|  | mem,A+CN | 0.197 | 0.061 | 3.221 | 0.158 |
|  | mem,A+AD | -0.440 | 0.073 | -6.054 | < .001 |
|  | mem,A+MCI | -0.468 | 0.031 | -14.912 | < .001 |
|  | mem,HC | 0.300 | 0.052 | 5.755 | < .001 |
|  | vs,A+CN | 0.723 | 0.061 | 11.815 | < .001 |
|  | vs,A+AD | 0.347 | 0.073 | 4.772 | < .001 |
|  | vs,A+MCI | 0.422 | 0.031 | 13.423 | < .001 |
|  | vs,HC | 0.754 | 0.052 | 14.447 | < .001 |
| lan,HC | mem,A+CN | -0.325 | 0.060 | -5.407 | < .001 |
|  | mem,A+AD | -0.962 | 0.072 | -13.412 | < .001 |
|  | mem,A+MCI | -0.990 | 0.052 | -18.972 | < .001 |
|  | mem,HC | -0.222 | 0.030 | -7.414 | < .001 |
|  | vs,A+CN | 0.201 | 0.060 | 3.347 | 0.102 |
|  | vs,A+AD | -0.175 | 0.072 | -2.444 | 1.000 |
|  | vs,A+MCI | -0.100 | 0.052 | -1.923 | 1.000 |
|  | vs,HC | 0.232 | 0.030 | 7.770 | < .001 |
| mem,A+CN | mem,A+AD | -0.637 | 0.078 | -8.114 | < .001 |
|  | mem,A+MCI | -0.665 | 0.061 | -10.874 | < .001 |
|  | mem,HC | 0.103 | 0.060 | 1.719 | 1.000 |
|  | vs,A+CN | 0.526 | 0.040 | 13.161 | < .001 |
|  | vs,A+AD | 0.150 | 0.078 | 1.905 | 1.000 |
|  | vs,A+MCI | 0.224 | 0.061 | 3.668 | 0.031 |
|  | vs,HC | 0.557 | 0.060 | 9.271 | < .001 |
| mem,A+AD | mem,A+MCI | -0.029 | 0.073 | -0.393 | 1.000 |
|  | mem,HC | 0.740 | 0.072 | 10.323 | < .001 |
|  | vs,A+CN | 1.163 | 0.078 | 14.813 | < .001 |
|  | vs,A+AD | 0.786 | 0.052 | 15.267 | < .001 |
|  | vs,A+MCI | 0.861 | 0.073 | 11.857 | < .001 |
|  | vs,HC | 1.194 | 0.072 | 16.650 | < .001 |
| mem,A+MCI | mem,HC | 0.769 | 0.052 | 14.728 | < .001 |
|  | vs,A+CN | 1.191 | 0.061 | 19.468 | < .001 |
|  | vs,A+AD | 0.815 | 0.073 | 11.219 | < .001 |
|  | vs,A+MCI | 0.890 | 0.031 | 28.335 | < .001 |
|  | vs,HC | 1.222 | 0.052 | 23.420 | < .001 |
| mem,HC | vs,A+CN | 0.423 | 0.060 | 7.034 | < .001 |
|  | vs,A+AD | 0.046 | 0.072 | 0.645 | 1.000 |
|  | vs,A+MCI | 0.121 | 0.052 | 2.321 | 1.000 |
|  | vs,HC | 0.454 | 0.030 | 15.184 | < .001 |
| vs,A+CN | vs,A+AD | -0.376 | 0.078 | -4.794 | < .001 |
|  | vs,A+MCI | -0.301 | 0.061 | -4.926 | < .001 |
|  | vs,HC | 0.031 | 0.060 | 0.518 | 1.000 |
| vs,A+AD | vs,A+MCI | 0.075 | 0.073 | 1.031 | 1.000 |
|  | vs,HC | 0.407 | 0.072 | 5.682 | < .001 |
| vs,A+MCI | vs,HC | 0.333 | 0.052 | 6.371 | < .001 |
| Note.  Bonferroni adjusted confidence intervals. | | | | | |

| Table S2. Post Hoc Comparisons – ECog-StudyPartner ✻ groupe | | | | | |
| --- | --- | --- | --- | --- | --- |
|  |  | Mean Difference | SE | t | p _bonf_ |
| ef,A+CN | ef,A+AD | -1.626 | 0.080 | -20.388 | < .001 |
|  | ef,A+MCI | -0.545 | 0.062 | -8.767 | < .001 |
|  | ef,HC | 0.009 | 0.061 | 0.155 | 1.000 |
|  | lan,A+CN | 0.062 | 0.041 | 1.513 | 1.000 |
|  | lan,A+AD | -1.388 | 0.080 | -17.410 | < .001 |
|  | lan,A+MCI | -0.524 | 0.062 | -8.431 | < .001 |
|  | lan,HC | 0.049 | 0.061 | 0.808 | 1.000 |
|  | mem,A+CN | -0.145 | 0.041 | -3.534 | 0.050 |
|  | mem,A+AD | -2.102 | 0.080 | -26.352 | < .001 |
|  | mem,A+MCI | -1.084 | 0.062 | -17.439 | < .001 |
|  | mem,HC | -0.142 | 0.061 | -2.323 | 1.000 |
|  | vs,A+CN | 0.120 | 0.041 | 2.937 | 0.403 |
|  | vs,A+AD | -1.224 | 0.080 | -15.345 | < .001 |
|  | vs,A+MCI | -0.297 | 0.062 | -4.774 | < .001 |
|  | vs,HC | 0.119 | 0.061 | 1.946 | 1.000 |
| ef,A+AD | ef,A+MCI | 1.081 | 0.074 | 14.646 | < .001 |
|  | ef,HC | 1.635 | 0.073 | 22.450 | < .001 |
|  | lan,A+CN | 1.688 | 0.080 | 21.167 | < .001 |
|  | lan,A+AD | 0.238 | 0.053 | 4.491 | < .001 |
|  | lan,A+MCI | 1.102 | 0.074 | 14.929 | < .001 |
|  | lan,HC | 1.675 | 0.073 | 22.997 | < .001 |
|  | mem,A+CN | 1.481 | 0.080 | 18.571 | < .001 |
|  | mem,A+AD | -0.476 | 0.053 | -8.994 | < .001 |
|  | mem,A+MCI | 0.542 | 0.074 | 7.341 | < .001 |
|  | mem,HC | 1.484 | 0.073 | 20.373 | < .001 |
|  | vs,A+CN | 1.746 | 0.080 | 21.899 | < .001 |
|  | vs,A+AD | 0.402 | 0.053 | 7.605 | < .001 |
|  | vs,A+MCI | 1.329 | 0.074 | 18.009 | < .001 |
|  | vs,HC | 1.745 | 0.073 | 23.950 | < .001 |
| ef,A+MCI | ef,HC | 0.555 | 0.053 | 10.457 | < .001 |
|  | lan,A+CN | 0.607 | 0.062 | 9.765 | < .001 |
|  | lan,A+AD | -0.843 | 0.074 | -11.428 | < .001 |
|  | lan,A+MCI | 0.021 | 0.032 | 0.648 | 1.000 |
|  | lan,HC | 0.594 | 0.053 | 11.208 | < .001 |
|  | mem,A+CN | 0.400 | 0.062 | 6.435 | < .001 |
|  | mem,A+AD | -1.557 | 0.074 | -21.090 | < .001 |
|  | mem,A+MCI | -0.539 | 0.032 | -16.722 | < .001 |
|  | mem,HC | 0.403 | 0.053 | 7.604 | < .001 |
|  | vs,A+CN | 0.666 | 0.062 | 10.704 | < .001 |
|  | vs,A+AD | -0.679 | 0.074 | -9.196 | < .001 |
|  | vs,A+MCI | 0.248 | 0.032 | 7.698 | < .001 |
|  | vs,HC | 0.664 | 0.053 | 12.517 | < .001 |
| ef,HC | lan,A+CN | 0.053 | 0.061 | 0.861 | 1.000 |
|  | lan,A+AD | -1.398 | 0.073 | -19.190 | < .001 |
|  | lan,A+MCI | -0.534 | 0.053 | -10.063 | < .001 |
|  | lan,HC | 0.040 | 0.031 | 1.298 | 1.000 |
|  | mem,A+CN | -0.154 | 0.061 | -2.530 | 1.000 |
|  | mem,A+AD | -2.111 | 0.073 | -28.979 | < .001 |
|  | mem,A+MCI | -1.094 | 0.053 | -20.624 | < .001 |
|  | mem,HC | -0.151 | 0.031 | -4.931 | < .001 |
|  | vs,A+CN | 0.111 | 0.061 | 1.818 | 1.000 |
|  | vs,A+AD | -1.233 | 0.073 | -16.929 | < .001 |
|  | vs,A+MCI | -0.306 | 0.053 | -5.776 | < .001 |
|  | vs,HC | 0.109 | 0.031 | 3.562 | 0.045 |
| lan,A+CN | lan,A+AD | -1.451 | 0.080 | -18.189 | < .001 |
|  | lan,A+MCI | -0.586 | 0.062 | -9.429 | < .001 |
|  | lan,HC | -0.013 | 0.061 | -0.209 | 1.000 |
|  | mem,A+CN | -0.207 | 0.041 | -5.046 | < .001 |
|  | mem,A+AD | -2.164 | 0.080 | -27.130 | < .001 |
|  | mem,A+MCI | -1.146 | 0.062 | -18.437 | < .001 |
|  | mem,HC | -0.204 | 0.061 | -3.340 | 0.104 |
|  | vs,A+CN | 0.058 | 0.041 | 1.424 | 1.000 |
|  | vs,A+AD | -1.286 | 0.080 | -16.123 | < .001 |
|  | vs,A+MCI | -0.359 | 0.062 | -5.773 | < .001 |
|  | vs,HC | 0.057 | 0.061 | 0.929 | 1.000 |
| lan,A+AD | lan,A+MCI | 0.864 | 0.074 | 11.711 | < .001 |
|  | lan,HC | 1.438 | 0.073 | 19.736 | < .001 |
|  | mem,A+CN | 1.244 | 0.080 | 15.593 | < .001 |
|  | mem,A+AD | -0.713 | 0.053 | -13.485 | < .001 |
|  | mem,A+MCI | 0.304 | 0.074 | 4.123 | 0.005 |
|  | mem,HC | 1.247 | 0.073 | 17.113 | < .001 |
|  | vs,A+CN | 1.509 | 0.080 | 18.921 | < .001 |
|  | vs,A+AD | 0.165 | 0.053 | 3.114 | 0.225 |
|  | vs,A+MCI | 1.092 | 0.074 | 14.791 | < .001 |
|  | vs,HC | 1.507 | 0.073 | 20.690 | < .001 |
| lan,A+MCI | lan,HC | 0.573 | 0.053 | 10.814 | < .001 |
|  | mem,A+CN | 0.379 | 0.062 | 6.100 | < .001 |
|  | mem,A+AD | -1.577 | 0.074 | -21.373 | < .001 |
|  | mem,A+MCI | -0.560 | 0.032 | -17.370 | < .001 |
|  | mem,HC | 0.382 | 0.053 | 7.211 | < .001 |
|  | vs,A+CN | 0.645 | 0.062 | 10.369 | < .001 |
|  | vs,A+AD | -0.700 | 0.074 | -9.479 | < .001 |
|  | vs,A+MCI | 0.227 | 0.032 | 7.051 | < .001 |
|  | vs,HC | 0.643 | 0.053 | 12.123 | < .001 |
| lan,HC | mem,A+CN | -0.194 | 0.061 | -3.183 | 0.180 |
|  | mem,A+AD | -2.151 | 0.073 | -29.525 | < .001 |
|  | mem,A+MCI | -1.134 | 0.053 | -21.375 | < .001 |
|  | mem,HC | -0.191 | 0.031 | -6.229 | < .001 |
|  | vs,A+CN | 0.071 | 0.061 | 1.166 | 1.000 |
|  | vs,A+AD | -1.273 | 0.073 | -17.476 | < .001 |
|  | vs,A+MCI | -0.346 | 0.053 | -6.527 | < .001 |
|  | vs,HC | 0.069 | 0.031 | 2.264 | 1.000 |
| mem,A+CN | mem,A+AD | -1.957 | 0.080 | -24.535 | < .001 |
|  | mem,A+MCI | -0.939 | 0.062 | -15.107 | < .001 |
|  | mem,HC | 0.003 | 0.061 | 0.052 | 1.000 |
|  | vs,A+CN | 0.265 | 0.041 | 6.470 | < .001 |
|  | vs,A+AD | -1.079 | 0.080 | -13.528 | < .001 |
|  | vs,A+MCI | -0.152 | 0.062 | -2.443 | 1.000 |
|  | vs,HC | 0.264 | 0.061 | 4.320 | 0.002 |
| mem,A+AD | mem,A+MCI | 1.017 | 0.074 | 13.785 | < .001 |
|  | mem,HC | 1.960 | 0.073 | 26.902 | < .001 |
|  | vs,A+CN | 2.222 | 0.080 | 27.863 | < .001 |
|  | vs,A+AD | 0.878 | 0.053 | 16.599 | < .001 |
|  | vs,A+MCI | 1.805 | 0.074 | 24.454 | < .001 |
|  | vs,HC | 2.220 | 0.073 | 30.479 | < .001 |
| mem,A+MCI | mem,HC | 0.942 | 0.053 | 17.771 | < .001 |
|  | vs,A+CN | 1.205 | 0.062 | 19.377 | < .001 |
|  | vs,A+AD | -0.140 | 0.074 | -1.891 | 1.000 |
|  | vs,A+MCI | 0.787 | 0.032 | 24.420 | < .001 |
|  | vs,HC | 1.203 | 0.053 | 22.684 | < .001 |
| mem,HC | vs,A+CN | 0.262 | 0.061 | 4.297 | 0.002 |
|  | vs,A+AD | -1.082 | 0.073 | -14.853 | < .001 |
|  | vs,A+MCI | -0.155 | 0.053 | -2.924 | 0.424 |
|  | vs,HC | 0.261 | 0.031 | 8.493 | < .001 |
| vs,A+CN | vs,A+AD | -1.344 | 0.080 | -16.856 | < .001 |
|  | vs,A+MCI | -0.417 | 0.062 | -6.712 | < .001 |
|  | vs,HC | -0.002 | 0.061 | -0.028 | 1.000 |
| vs,A+AD | vs,A+MCI | 0.927 | 0.074 | 12.560 | < .001 |
|  | vs,HC | 1.343 | 0.073 | 18.429 | < .001 |
| vs,A+MCI | vs,HC | 0.416 | 0.053 | 7.837 | < .001 |
| Note.  Bonferroni adjusted confidence intervals. | | | | | |

| Table S3. Post Hoc Comparisons - ACD ✻ groupe | | | | | |
| --- | --- | --- | --- | --- | --- |
|  |  | Mean Difference | SE | t | p _bonf_ |
| ef,A+CN | ef,A+AD | 1.232 | 0.098 | 12.518 | < .001 |
|  | ef,A+MCI | 0.195 | 0.077 | 2.547 | 1.000 |
|  | ef,HC | 0.025 | 0.075 | 0.333 | 1.000 |
|  | lan,A+CN | -0.231 | 0.048 | -4.777 | < .001 |
|  | lan,A+AD | 0.901 | 0.098 | 9.156 | < .001 |
|  | lan,A+MCI | -0.064 | 0.077 | -0.829 | 1.000 |
|  | lan,HC | -0.115 | 0.075 | -1.529 | 1.000 |
|  | mem,A+CN | -0.246 | 0.048 | -5.077 | < .001 |
|  | mem,A+AD | 1.074 | 0.098 | 10.917 | < .001 |
|  | mem,A+MCI | 0.028 | 0.077 | 0.367 | 1.000 |
|  | mem,HC | -0.146 | 0.075 | -1.934 | 1.000 |
|  | vs,A+CN | 0.015 | 0.048 | 0.304 | 1.000 |
|  | vs,A+AD | 0.983 | 0.098 | 9.988 | < .001 |
|  | vs,A+MCI | 0.131 | 0.077 | 1.703 | 1.000 |
|  | vs,HC | 0.048 | 0.075 | 0.632 | 1.000 |
| ef,A+AD | ef,A+MCI | -1.036 | 0.091 | -11.381 | < .001 |
|  | ef,HC | -1.206 | 0.090 | -13.425 | < .001 |
|  | lan,A+CN | -1.463 | 0.098 | -14.868 | < .001 |
|  | lan,A+AD | -0.331 | 0.062 | -5.301 | < .001 |
|  | lan,A+MCI | -1.295 | 0.091 | -14.225 | < .001 |
|  | lan,HC | -1.347 | 0.090 | -14.985 | < .001 |
|  | mem,A+CN | -1.477 | 0.098 | -15.015 | < .001 |
|  | mem,A+AD | -0.157 | 0.062 | -2.524 | 1.000 |
|  | mem,A+MCI | -1.203 | 0.091 | -13.218 | < .001 |
|  | mem,HC | -1.377 | 0.090 | -15.324 | < .001 |
|  | vs,A+CN | -1.217 | 0.098 | -12.368 | < .001 |
|  | vs,A+AD | -0.249 | 0.062 | -3.988 | 0.008 |
|  | vs,A+MCI | -1.101 | 0.091 | -12.092 | < .001 |
|  | vs,HC | -1.184 | 0.090 | -13.174 | < .001 |
| ef,A+MCI | ef,HC | -0.170 | 0.065 | -2.602 | 1.000 |
|  | lan,A+CN | -0.427 | 0.077 | -5.561 | < .001 |
|  | lan,A+AD | 0.705 | 0.091 | 7.748 | < .001 |
|  | lan,A+MCI | -0.259 | 0.038 | -6.806 | < .001 |
|  | lan,HC | -0.310 | 0.065 | -4.746 | < .001 |
|  | mem,A+CN | -0.441 | 0.077 | -5.750 | < .001 |
|  | mem,A+AD | 0.879 | 0.091 | 9.652 | < .001 |
|  | mem,A+MCI | -0.167 | 0.038 | -4.395 | 0.001 |
|  | mem,HC | -0.341 | 0.065 | -5.211 | < .001 |
|  | vs,A+CN | -0.181 | 0.077 | -2.355 | 1.000 |
|  | vs,A+AD | 0.787 | 0.091 | 8.648 | < .001 |
|  | vs,A+MCI | -0.065 | 0.038 | -1.701 | 1.000 |
|  | vs,HC | -0.148 | 0.065 | -2.259 | 1.000 |
| ef,HC | lan,A+CN | -0.256 | 0.075 | -3.404 | 0.083 |
|  | lan,A+AD | 0.876 | 0.090 | 9.744 | < .001 |
|  | lan,A+MCI | -0.089 | 0.065 | -1.355 | 1.000 |
|  | lan,HC | -0.140 | 0.036 | -3.874 | 0.013 |
|  | mem,A+CN | -0.271 | 0.075 | -3.596 | 0.041 |
|  | mem,A+AD | 1.049 | 0.090 | 11.672 | < .001 |
|  | mem,A+MCI | 0.003 | 0.065 | 0.047 | 1.000 |
|  | mem,HC | -0.171 | 0.036 | -4.715 | < .001 |
|  | vs,A+CN | -0.010 | 0.075 | -0.137 | 1.000 |
|  | vs,A+AD | 0.958 | 0.090 | 10.655 | < .001 |
|  | vs,A+MCI | 0.106 | 0.065 | 1.613 | 1.000 |
|  | vs,HC | 0.022 | 0.036 | 0.621 | 1.000 |
| lan,A+CN | lan,A+AD | 1.132 | 0.098 | 11.506 | < .001 |
|  | lan,A+MCI | 0.168 | 0.077 | 2.185 | 1.000 |
|  | lan,HC | 0.116 | 0.075 | 1.541 | 1.000 |
|  | mem,A+CN | -0.014 | 0.048 | -0.300 | 1.000 |
|  | mem,A+AD | 1.305 | 0.098 | 13.267 | < .001 |
|  | mem,A+MCI | 0.259 | 0.077 | 3.381 | 0.090 |
|  | mem,HC | 0.086 | 0.075 | 1.137 | 1.000 |
|  | vs,A+CN | 0.246 | 0.048 | 5.082 | < .001 |
|  | vs,A+AD | 1.214 | 0.098 | 12.338 | < .001 |
|  | vs,A+MCI | 0.362 | 0.077 | 4.718 | < .001 |
|  | vs,HC | 0.279 | 0.075 | 3.702 | 0.027 |
| lan,A+AD | lan,A+MCI | -0.964 | 0.091 | -10.592 | < .001 |
|  | lan,HC | -1.016 | 0.090 | -11.304 | < .001 |
|  | mem,A+CN | -1.146 | 0.098 | -11.653 | < .001 |
|  | mem,A+AD | 0.173 | 0.062 | 2.777 | 0.664 |
|  | mem,A+MCI | -0.873 | 0.091 | -9.585 | < .001 |
|  | mem,HC | -1.046 | 0.090 | -11.643 | < .001 |
|  | vs,A+CN | -0.886 | 0.098 | -9.006 | < .001 |
|  | vs,A+AD | 0.082 | 0.062 | 1.313 | 1.000 |
|  | vs,A+MCI | -0.770 | 0.091 | -8.459 | < .001 |
|  | vs,HC | -0.853 | 0.090 | -9.494 | < .001 |
| lan,A+MCI | lan,HC | -0.052 | 0.065 | -0.788 | 1.000 |
|  | mem,A+CN | -0.182 | 0.077 | -2.374 | 1.000 |
|  | mem,A+AD | 1.138 | 0.091 | 12.495 | < .001 |
|  | mem,A+MCI | 0.092 | 0.038 | 2.411 | 1.000 |
|  | mem,HC | -0.082 | 0.065 | -1.253 | 1.000 |
|  | vs,A+CN | 0.078 | 0.077 | 1.021 | 1.000 |
|  | vs,A+AD | 1.046 | 0.091 | 11.492 | < .001 |
|  | vs,A+MCI | 0.194 | 0.038 | 5.105 | < .001 |
|  | vs,HC | 0.111 | 0.065 | 1.699 | 1.000 |
| lan,HC | mem,A+CN | -0.131 | 0.075 | -1.734 | 1.000 |
|  | mem,A+AD | 1.189 | 0.090 | 13.233 | < .001 |
|  | mem,A+MCI | 0.143 | 0.065 | 2.190 | 1.000 |
|  | mem,HC | -0.030 | 0.036 | -0.841 | 1.000 |
|  | vs,A+CN | 0.130 | 0.075 | 1.725 | 1.000 |
|  | vs,A+AD | 1.098 | 0.090 | 12.216 | < .001 |
|  | vs,A+MCI | 0.246 | 0.065 | 3.757 | 0.022 |
|  | vs,HC | 0.163 | 0.036 | 4.495 | < .001 |
| mem,A+CN | mem,A+AD | 1.320 | 0.098 | 13.415 | < .001 |
|  | mem,A+MCI | 0.274 | 0.077 | 3.570 | 0.045 |
|  | mem,HC | 0.100 | 0.075 | 1.330 | 1.000 |
|  | vs,A+CN | 0.260 | 0.048 | 5.381 | < .001 |
|  | vs,A+AD | 1.228 | 0.098 | 12.486 | < .001 |
|  | vs,A+MCI | 0.376 | 0.077 | 4.907 | < .001 |
|  | vs,HC | 0.293 | 0.075 | 3.895 | 0.013 |
| mem,A+AD | mem,A+MCI | -1.046 | 0.091 | -11.488 | < .001 |
|  | mem,HC | -1.220 | 0.090 | -13.572 | < .001 |
|  | vs,A+CN | -1.059 | 0.098 | -10.767 | < .001 |
|  | vs,A+AD | -0.091 | 0.062 | -1.465 | 1.000 |
|  | vs,A+MCI | -0.943 | 0.091 | -10.362 | < .001 |
|  | vs,HC | -1.027 | 0.090 | -11.422 | < .001 |
| mem,A+MCI | mem,HC | -0.174 | 0.065 | -2.655 | 0.966 |
|  | vs,A+CN | -0.013 | 0.077 | -0.174 | 1.000 |
|  | vs,A+AD | 0.955 | 0.091 | 10.484 | < .001 |
|  | vs,A+MCI | 0.102 | 0.038 | 2.694 | 0.855 |
|  | vs,HC | 0.019 | 0.065 | 0.297 | 1.000 |
| mem,HC | vs,A+CN | 0.160 | 0.075 | 2.129 | 1.000 |
|  | vs,A+AD | 1.128 | 0.090 | 12.555 | < .001 |
|  | vs,A+MCI | 0.276 | 0.065 | 4.222 | 0.003 |
|  | vs,HC | 0.193 | 0.036 | 5.336 | < .001 |
| vs,A+CN | vs,A+AD | 0.968 | 0.098 | 9.838 | < .001 |
|  | vs,A+MCI | 0.116 | 0.077 | 1.511 | 1.000 |
|  | vs,HC | 0.033 | 0.075 | 0.436 | 1.000 |
| vs,A+AD | vs,A+MCI | -0.852 | 0.091 | -9.359 | < .001 |
|  | vs,HC | -0.935 | 0.090 | -10.405 | < .001 |
| vs,A+MCI | vs,HC | -0.083 | 0.065 | -1.270 | 1.000 |
| Note.  Bonferroni adjusted confidence intervals. | | | | | |

| **Figure S3. Correlations between subjective and objective cognitive measures** |
| --- |
| 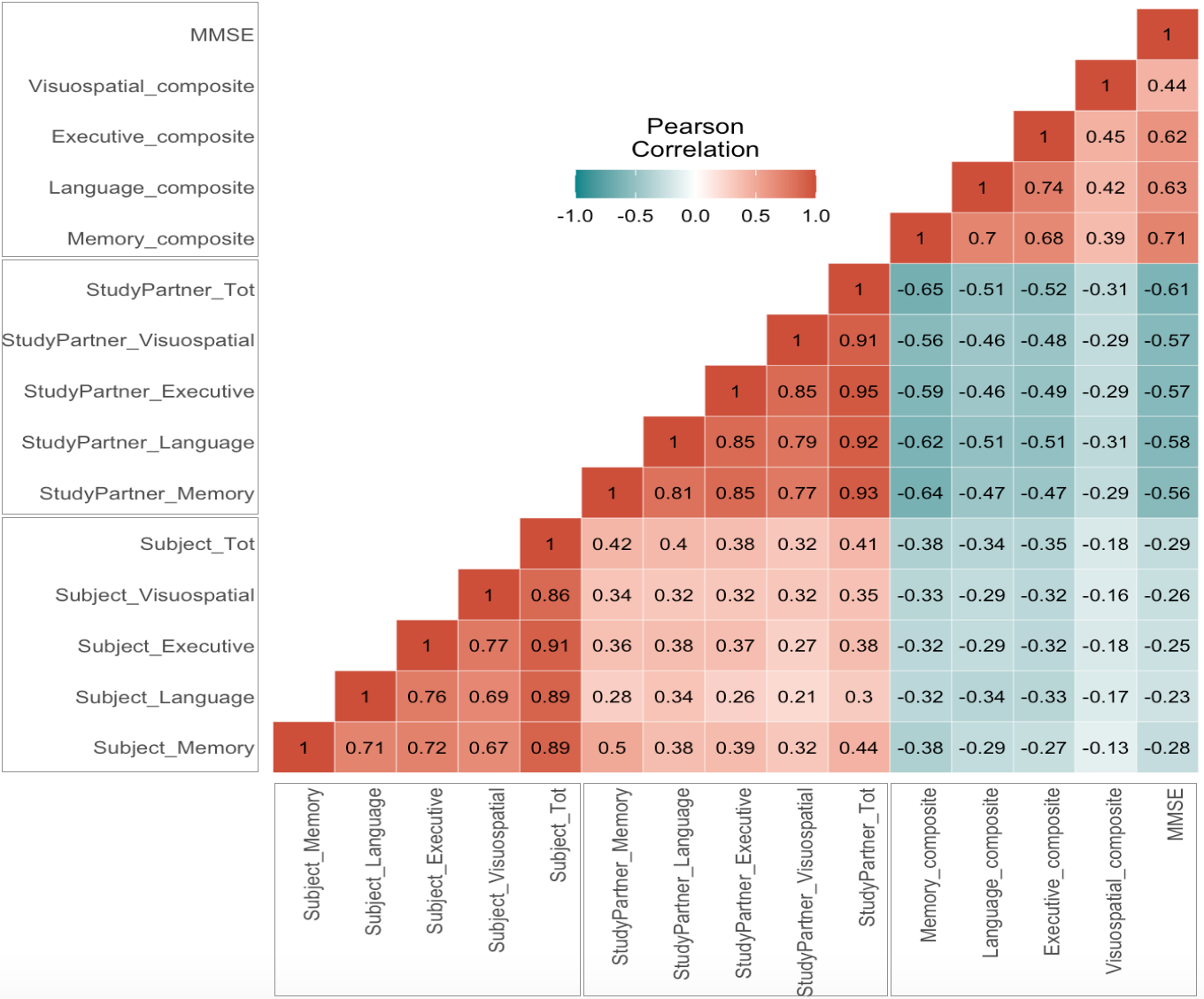 |
